# A DNA barcode library for woody plants in tropical and subtropical China

**DOI:** 10.1038/s41597-023-02742-7

**Published:** 2023-11-22

**Authors:** Lu Jin, Hao-You Shi, Ting Li, Nan Zhao, Yong Xu, Tian-Wen Xiao, Feng Song, Chen-Xin Ma, Qiao-Ming Li, Lu-Xiang Lin, Xiao-Na Shao, Bu-Hang Li, Xiang-Cheng Mi, Hai-Bao Ren, Xiu-Juan Qiao, Ju-Yu Lian, Hu Du, Xue-Jun Ge

**Affiliations:** 1 Key Laboratory of Plant Resources Conservation and Sustainable Utilization, South China Botanical Garden, Chinese Academy of Sciences, Guangzhou, 510650 China; 2 Central South Academy of Inventory and Planning of NFGA, Changsha, 410014 China; 3 Yiyang Forestry Bureau, Yiyang, 413000 China; 4 Hunan Police Academy, Changsha, 410138 China; 5 Conghua Middle School, Guangzhou, 510900 China; 6 College of Forestry, Central South University of Forestry & Technology, Changsha, 410004 China; 7 CAS Key Laboratory of Tropical Forest Ecology, Xishuangbanna Tropical Botanical Garden, Chinese Academy of Sciences, Kunming, 650201 China; 8 School of Life Sciences, Sun Yat-Sen University, Guangzhou, 510275 China; 9 State Key Laboratory of Vegetation and Environmental Change, Institute of Botany, Chinese Academy of Sciences, Beijing, 100093 China; 10 Key Laboratory of Aquatic Botany and Watershed Ecology, Wuhan Botanical Garden, Chinese Academy of Sciences, Wuhan, 430074 China; 11 Center of Conservation Biology, Core Botanical Gardens, Chinese Academy of Sciences, Wuhan, 430074 China; 12 Center of Plant Ecology, Core Botanical Gardens, Chinese Academy of Sciences, Guangzhou, 510650 China; 13 Institute of Subtropical Agriculture, Chinese Academy of Sciences, Changsha, Hunan 410125 China

**Keywords:** Genetic markers, Taxonomy

## Abstract

The application of DNA barcoding has been significantly limited by the scarcity of reliable specimens and inadequate coverage and replication across all species. The deficiency of DNA barcode reference coverage is particularly striking for highly biodiverse subtropical and tropical regions. In this study, we present a comprehensive barcode library for woody plants in tropical and subtropical China. Our dataset includes a standard barcode library comprising the four most widely used barcodes (*rbcL*, *matK*, ITS, and ITS2) for 2,520 species from 4,654 samples across 49 orders, 144 families, and 693 genera, along with 79 samples identified at the genus level. This dataset also provides a super-barcode library consisting of 1,239 samples from 1,139 species, 411 genera, 113 families, and 40 orders. This newly developed library will serve as a valuable resource for DNA barcoding research in tropical and subtropical China and bordering countries, enable more accurate species identification, and contribute to the conservation and management of tropical and subtropical forests.

## Background & Summary

Accurate species identification is crucial for biological research, particularly in the areas of biodiversity conservation and utilization. However, traditional morphology-based identification has significant limitations, including incorrect identifications, unrecognized cryptic species, the absence of diagnostic characters in specific developmental stages, and the need for specialized expertise^1^. Moreover, woody plant identification in tropical or subtropical regions poses a formidable challenge owing to the lack of access to reproductive organs necessary to differentiate similar species during field surveys^2^. To address these challenges, DNA barcoding has emerged as a powerful tool that can help circumvent the limitations of morphological identification^1,3^.

DNA barcodes are short standardized sequences that can be used to identify species based on materials from the entire organism, fragmented tissue, or even environmental DNA^4^. However, while *cytochrome c oxidase subunit 1* (CO1) performs well universally for animals, it is not appropriate for plants owing to the lower rates of divergence in plant compared to animal mitochondrial genomes^3^. The plant working group of the Consortium for the Barcoding of Life (CBOL) has recommended *rbcL* and *matK* as core barcodes for land plants after comparing the performance of 7 candidate plastid loci^5^. Further, the internal transcribed spacer (ITS) or ITS2 has been reported to have the highest degree of species discrimination for seed plants^6^. Based on these findings, plastid (*rbcL* and *matK*) and nuclear fragments (ITS/ITS2) have been widely used as standard DNA barcodes for plants.

Despite their wide application, standard DNA barcodes have insufficient variation, which limits their usefulness in identifying recently diverged and rapidly radiated groups^7,8^. To address this issue, the use of whole plastid genomes as super-barcodes has been proposed^9–11^. Ranging from 110 to 160 kbp, the plastid genome can provide more variation than standard DNA barcodes to distinguish closely related species, thus improving phylogenetic resolution at lower taxonomic levels in plant phylogenetic and population genetic analyses^11,12^. Genome skimming, a low-coverage shotgun sequencing approach, has been applied widely to obtain complete plastid genomes and high-copy nuclear ribosomal sequences (nrDNA)^13–16^. This method recovers all plastid loci and ITS simultaneously, which overcomes problems of low PCR efficiency and sequence retrieval for the standard barcode sequences, and contributes to the reference database for standard barcodes^10^.

The tropical and subtropical moist biomes of all continents have the highest tree species richness, with southeast Asia being one of the most diverse regions^17^. Within this region, China has exceptionally high biodiversity and endemism. The region of South-Central China is recognized as a hotspot for biodiversity but has experienced significant loss of habitats due to human activities^18^. According to the Atlas of Woody Plants in China^19^, there are 11,405 woody species in China, of which 244 (2.1%) are gymnosperms, 10,480 (91.9%) are dicots, and 664 (5.8%) are monocots. Woody plant species richness in China is concentrated primarily in the southern mountainous regions, which are dominated by subtropical evergreen broad-leaved and tropical monsoon rain forests. These regions include the south and southeast areas of Yunnan, mountains at the borders of Guangxi and Yunnan, and the Hengduan, Wuyi, Nanling, and Wuling Mountains.

Here, we developed a comprehensive barcode library that includes both standard barcodes and super-barcodes for woody plants in tropical and subtropical China. The standard barcode library contains the four most widely used barcodes (*rbcL*, *matK*, ITS, and ITS2) for 2,520 species from 4,733 samples across 49 orders, 144 families, and 683 genera, and includes 79 samples identified to the genus level, while the super-barcode library consists of 1,239 samples from 1,139 species, 411 genera, 113 families, and 40 orders. Our library generated 5,937 novel standard barcode sequences for 1,696 species and 262 new plastid genome sequences for 258 species that will enrich the current barcode database for woody plants in subtropical and tropical China. This barcode library represents a valuable resource for taxonomic identification, ecological and evolutionary research, and biodiversity conservation in subtropical and tropical China and bordering countries. Furthermore, by integrating this DNA barcode library with other datasets, such as datasets containing functional traits^20^ and geographic distribution information^19^, we can expand our comprehension of the evolutionary history and temporal dynamics of the flora in this region, which will provide valuable insights for conservation efforts in the face of global climate change^21^.

## Methods

### Sample collection and identification

**Fig. 1:**
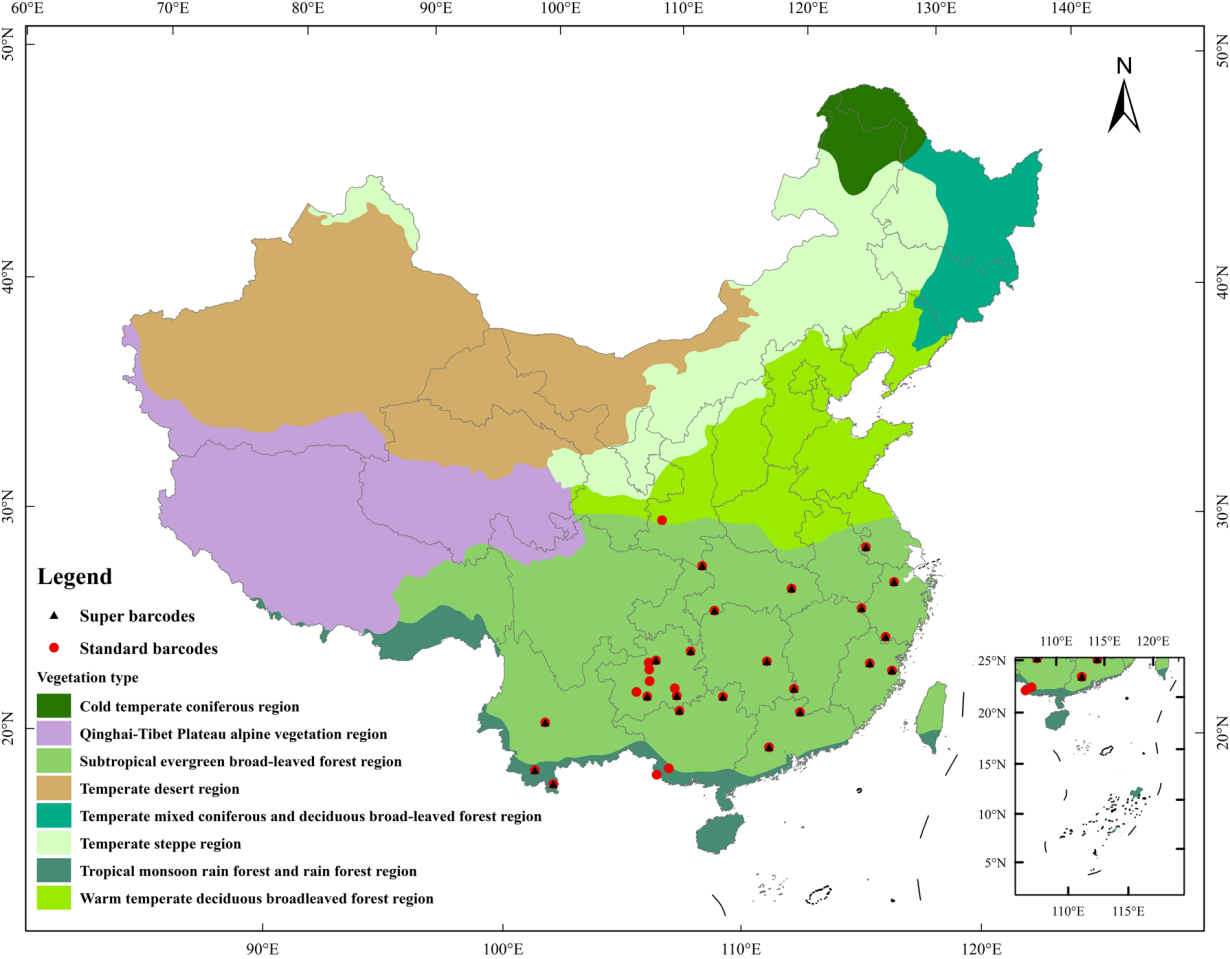
The localities we collected specimens in tropical and subtropical China. Red dots represent sample sites of standard barcodes, while black triangles represent sample sites of super barcodes.

To create a comprehensive library of standard barcodes and super-barcodes for woody plants in subtropical and tropical China, we conducted fieldwork in 11 provinces and 29 cities, representing a significant proportion of the plant diversity of tropical and subtropical China (Fig. 1, Table S1). The scientific names of species in our dataset were standardized with reference to The Plant List (http://www.theplantlist.org/) using the ‘status’ function of the R package ‘plantlist’ version 0.7.2^22^ and the Flora of China. For each species, one to nine individuals were sampled, and fresh leaf material was dried in silica gel for subsequent DNA extraction. Voucher specimens were identified by professional taxonomists using morphological characters and were deposited in the herbarium of the South China Botanical Garden (IBSC).

### DNA extraction, sequencing, and assembly

Total genomic DNA was isolated from silica-dried leaf tissue using the cetyltrimethylammonium bromide (CTAB) method^23^. Amplification of *rbcL* utilized one universal primer set (*rbcL*a-F/-R). The *matK* with low amplification success rate required three pairs of primers (Kim_3F/1 R, xF/5r, Gym_F1A/R1A), of which Gym_F1A/R1A^24^. For the ITS marker, two primer pairs (ITS-Leu/4, ITS5/4) were utilized initially; samples that failed to amplify were re-amplified for ITS2. The ITS2 was amplified by one universal primer set (ITS2_S2F/S3R). Each 25 μl PCR reaction mixture included 2.5 μl 10 × PCR Buffer, 2 μl dNTPs (2.5 mM), 0.5 μl of each primer (10 μM), 2 μl DNA template, 0.2 μl rTaq (5U/μl), 0.5 μl DNA template, and 18.8 μl ddH_2_O. Mg^2+^ (5%) or dimethyl sulfoxide (DMSO) (5%) was added to improve the sequence recovery success rate of *matK* and ITS/ITS2. Mg^2+^ can act as a cofactor during polymerization^25^, and DMSO can resolve secondary DNA structures by binding the major and minor grooves of DNA strands^26^. The details of primers and references are shown in Table S2. All PCR products were sequenced using the Sanger sequencing method on an ABI 3730 DNA analyzer. All original trace files were assembled and checked using Geneious v11.0.2^27^.

For the samples collected for super-barcodes, we implemented the genome-skimming method to acquire complete plastid genome and nrDNA sequences. DNA extracts were sent to Beijing Genomics Institute (BGI, Shenzhen, China) for library preparation and genome-skimming sequencing. Following the MGIEasy Universal DNA Library Prep Set user manual v.1.0 (MGI Tech, https://en.mgi-tech.com/download/files.html), the DNA extracts were sheared into 300 to 500 bp fragments for library construction. Paired-end sequencing (2 × 150 bp) was performed on the Illumina HiSeq X Ten platform at BGI. Phred quality scores and %GC content of raw reads were determined using FastQC 0.11.5 (http://www.bioinformatics.babraham.ac.uk/projects/fastqc/). Subsequently, low-quality reads and adapters were removed using Trimmomatic v.0.35^28^, generating approximately 2–3 Gb of clean read data for each sample. The plastid genomes and nrDNA were *de novo* assembled from the clean read data using GetOrganelle v1.7.6^29^. Then, the plastomes were annotated using DOGMA^30^ and GeSeq^31^, with start and stop codons manually adjusted in Geneious v11.0.2. The nrDNAs were annotated using Geneious v11.0.2 as well. For subsequent barcode analyses, plastid markers (*rbcL* and *matK*) were extracted from plastomes, while ITS/ITS2 were extracted from nrDNA.

### Data verification

All the sequences obtained by Sanger sequencing were verified by using the BLASTn tool. If query sequences with top hits were from the same species or genus as the submitted sequences, they were retained for further analyses^28^. Sequences with conflicts between the search outcomes and taxonomic identification were examined carefully to determine whether there was contamination (e.g., mixed ITS sequences of insects and fungi), incorrect sequencing (e.g., mix-up of DNA samples), or incorrect identification (i.e., a mismatch between sequence Blast results and specimen identification). The contaminated or incorrect sequences were excluded from further analyses, while the samples with incorrect identifications were re-identified by taxonomic experts. However, 79 samples were not identified with certainty and thus were not included in further analyses. To minimize the impact of missing data, we only included species with samples from at least two individuals in our subsequent analyses.

We utilized three common methods to assess the discriminatory power of the four standard barcodes. First, genetic distances were used to identify the presence of “barcode gaps”, which occur when minimum inter-specific genetic distances are higher than maximum intra-specific genetic distances^32^. Following the methods of Gill *et al*.^33^, the uncorrected intra- and inter-specific genetic distances for each barcode separately and their combinations were calculated with the function ‘distancematrix’ in DECIPHER^34^. Second, we used TaxonDNA v1.8^35^ to perform identification based on genetic distances. For the “Best match” (BM) approach, an identification was considered successful if the query and its closest sequence matches were from the same species, while mismatched species were categorized as incorrect identifications. Results with matching multiple different species were considered ambiguous. For the ‘Best close match’ (BCM) method, a threshold value that was less than 95% of all intra-specific distances was established^35^. Queries without any sequence matches below the threshold were considered as unidentified, while correct, ambiguous, and incorrect identifications were defined as for the BM method. Third, we used a tree-based method, where species clustering in a monophyletic group was considered a successful resolution. We aligned the standard barcode sequences using MAFFT v7.4^36^ and adjusted them manually in Geneious v11.0.2. Alignment of *rbcL* and *matK* was performed with default parameters. ITS and ITS2 were aligned by families and then the sequences were concatenated. The gymnosperm sequences were removed to avoid inaccuracy of ITS alignment caused by the higher variation of internal transcribed spacer-1 (ITS1) in these species. We constructed Maximum-likelihood (ML) trees for each marker and their combinations using RAxML 8.2.12^37^ under the GTRGAMMA model. Node supports were evaluated with 1,000 bootstrap replicates, and monophyletic clades with support greater than or equal to 50% were defined as successful identifications^38^.

To confirm a higher phylogenetic resolution of the super-barcode in comparison to the standard barcode, we evaluated the node supports of the plastid genome tree. We extracted all protein-coding genes from the assembled plastid genomes using a python script (https://github.com/Kinggerm/PersonalUtilities). A total of 78 genes that occurred most frequently in all species were selected to construct a plastid genome tree. Sequences were aligned by MAFFT v7.4^36^ for each locus and then concatenated to generate a supermatrix. Model selection was performed using jModelTest v2.0^39^, and the maximum likelihood tree was constructed under the best model GTRGAMMA by RAxML 8.2.12^37^. To evaluate the node supports, 1000 bootstraps were replicated. As we had fewer replicated samples for super-barcodes, we did not test the resolution for super-barcodes to identify closely related species.

## Data Records

All standard and super-barcode sequences, sequence records, and specimen pictures from this study are stored at Figshare^40^. The raw reads data for all newly generated plastid genomes in this study have been deposited in the NCBI Sequence Read Archive (SRA) database under the accession numbers SRX22362678^41^-SRX22362939^42^. We successfully generated standard barcodes for 1,696 species from 2,524 individuals across 48 orders, 130 families, and 547 genera. In addition, we identified 79 samples at the genus level. We also incorporated partial standard barcode data from our previous study on Dinghushan National Nature Reserve^43^, which included 517 woody species from 969 samples. Furthermore, we extracted *rbcL*, *matK*, and ITS/ITS2 from our plastid genome dataset (see below). Overall, we constructed a standard barcode library containing 2,520 species from 4,733 samples across 49 orders, 144 families, and 683 genera. This library, which also includes 79 samples currently identified to the genus level, comprises a total of 15,090 accessions for the four most commonly used barcodes (*rbcL*, *matK*, ITS, and ITS2). Thus, for the standard barcode library, we obtained 2,520 species from 4,654 individuals, resulting in a total of 14,837 sequences^40^. Specifically, we acquired 4,451 *rbcL* sequences, 4,055 *matK* sequences, 2,905 ITS sequences, and 3,426 ITS2 sequences (Table 1). These sequences cover 683 genera, 144 families, and 49 orders of woody plants in tropical and subtropical China.

**Table 1:** Summary of standard barcodes for woody plants in tropical and subtropical China (without sp.).

Sequence information	Barcodes			
	*rbcL*	*matK*	ITS	ITS2
Number of sampled families	144	140	128	133
Number of sampled genera	676	650	580	624
Number of sampled species	2462	2321	1865	2066
Number of sampled individuals	4451	4055	2905	3426
Percentage of species with multiple individuals	1009/2462	905/2321	615/1865	754/2066
	40.98%	38.99%	32.98%	36.50%

For super-barcodes, 971 plastid genomes were obtained from our previous research^44,45^. In addition, 262 plastid genomes belonging to 71 families, 170 genera, and 258 species were newly generated in the present study. Finally, the super-barcode library included 1,239 samples belonging to 40 orders, 113 families, 411 genera, and 1,139 species^40^.

The sequence records file has two separate sheets for standard and super-barcode libraries. Each record in the list for super-barcodes contains (1) associated species information including sample ID, order, family, genus, and species; (2) sequence information including GenBank accession numbers and the presence or absence of the four standard barcodes; and (3) specimen information including collection sites, latitude and longitude, elevation, collectors, collection date, identifier, museum ID, and the storing institution. The list for standard barcodes contains additional information including BOLD ID, sequence length, trace count, and image count. Moreover, all specimen details and standard DNA barcode sequences were uploaded to the BOLD system, which is open to the public, in the dataset “DS-EBLF” (10.5883/DS-EBLF).

## Technical Validation

**Fig. 2:**
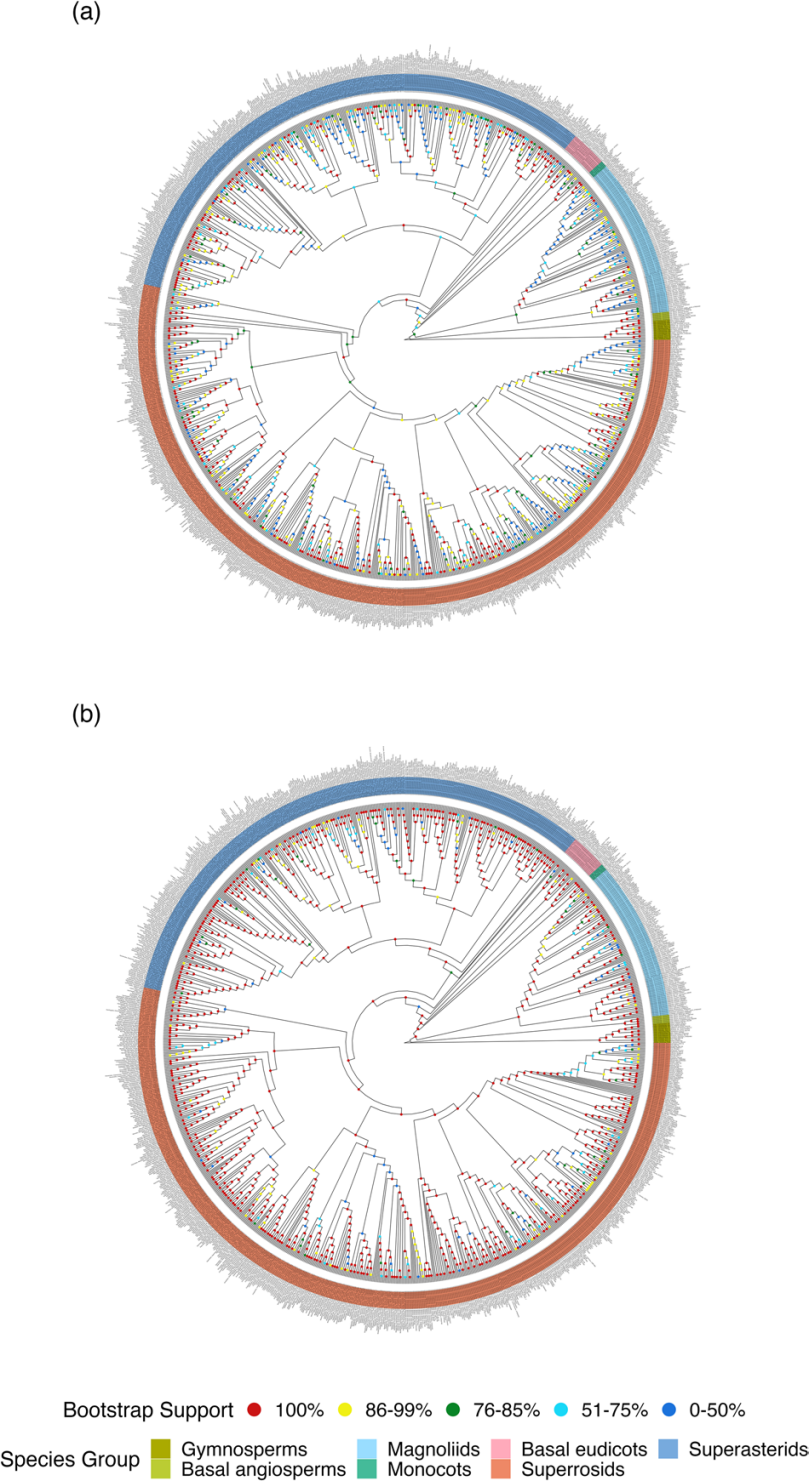
The phylogenies constructed for 1,139 woody species of 1,239 samples using super barcodes and extracted standard barcodes in the tropical and subtropical China. (**a**) the standard barcode tree constructed with three standard barcodes (*rbcL*, *matK* and ITS) and (**b**) plastid genome tree constructed with 78 protein-coding genes from plastid genomes. Dots of different colors in the nodes represent the different levels of bootstrap supports. Different colored strips represent seven major taxonomic groups (Gymnosperms, basal angiosperms, Magnoliids, Monocots, basal eudicots, Superrosids, and Superasterids).

The discriminatory power of the standard barcodes among species were evaluated with multiple individuals using three common methods (Table 2). The results of the distance-based “BM/BCM” method demonstrated that BM and BCM had almost the same correct, ambiguous, and incorrect identification rate for all barcodes, with BM having slightly higher rates than BCM. ITS had the highest correct identifications (72.66% for BCM) while the resolution for *rbcL* and *matK* was lower with higher ambiguous identification (Table 2). The combination RMI had the highest species resolution for the barcoding gap and tree-based method (59.07% and 66.61%, respectively) (Table 2). While *rbcL* and *matK* had the lowest resolution for the data set with abundant species, ITS performed best for the four single barcodes using the three methods (71.68%/72.66%, 58.05%, and 61.33% for BM/BCM, the barcoding gap, and tree-based method, respectively) (Table 2), which is consistent with previous DNA barcode studies (e.g., Hu *et al*.^38^; Liu *et al*.^43^; Gill *et al*.^33^; Huang *et al*.^2^). Moreover, we observed significant improvements in node supports for the plastid genome tree compared to the standard barcode tree, particularly for species-rich families (Fig. 2, Table 3). In the standard barcode tree, 20.44% of the nodes showed low bootstrap support values (0 < BS < 50), and only 57.27% of the nodes had high bootstrap support values (BS > 85). In contrast, in the plastid genome tree, 5.49% of the nodes had low bootstrap support values, and 85.86% of the nodes had high bootstrap support values (Fig. 2, Table 3). Both the standard barcode tree and the plastid genome tree can be found on Figshare^40^.

**Table 2:** Species identification rates for standard barcodes based on three methods.

	BM/BCM (taxonDNA)			Barcoding gap	Tree-based
	Correct identifications	Ambiguous	Incorrect identifications	with gap (%)	Species identification (%)
*rbcL*	37.02/37.55	53.82/53.95	8.02/8.48	30.53	25.30
*matK*	48.36/49.07	39.81/39.96	10.31/10.95	38.85	30.55
ITS	71.68/72.66	9.23/9.35	16.88/17.98	58.05	61.33
ITS2	66.31/67.12	15.98/16.08	15.79/16.79	51.19	42.82
RMI^*^	55.82/55.20	1.84/1.84	42.32/41.64	59.07	66.61

^*^the combination of *rbcL*, *matK*, and ITS2.

**Table 3:** Comparisons of bootstrap values for total and the most ten families sampled between the standard barcode tree and the plastid genome tree.

Clades (n=)	Phylogeny	Resolution			
		0%–50%	51%–70%	71%–85%	86%–100%
all (1239)	Standard barcode tree	20.44	13.17	8.97	57.27
	Plastid genome tree	5.49	5.01	3.47	85.86
Lauraceae (87)	Standard barcode tree	40.23	22.99	5.75	31.03
	Plastid genome tree	10.34	11.49	5.75	72.41
Rosaceae (65)	Standard barcode tree	21.54	16.92	12.31	49.23
	Plastid genome tree	6.15	6.15	9.23	78.46
Rubiaceae (57)	Standard barcode tree	7.02	7.02	12.28	73.68
	Plastid genome tree	3.51	0	5.26	91.23
Moraceae (54)	Standard barcode tree	22.22	14.81	11.11	51.85
	Plastid genome tree	6	0	0	94
Fabaceae (50)	Standard barcode tree	4	14	8	74
	Plastid genome tree	6	0	0	94
Fagaceae (47)	Standard barcode tree	44.68	14.89	19.15	21.28
	Plastid genome tree	10.64	12.77	6.38	70.21
Euphorbiaceae (42)	Standard barcode tree	23.81	4.76	9.52	61.9
	Plastid genome tree	7.14	11.9	2.38	78.57
Malvaceae (41)	Standard barcode tree	19.51	9.76	4.88	65.85
	Plastid genome tree	2.44	2.44	0	95.12
Aquifoliaceae (36)	Standard barcode tree	50	13.89	8.33	27.78
	Plastid genome tree	8.33	16.67	0	75
Sapindaceae (33)	Standard barcode tree	15.15	18.18	6.06	60.61
	Plastid genome tree	0	3.03	3.03	93.94

### Supplementary information

Supplementary Table

## Data Availability

The code used to check species names can be found in the R package ‘plantlist’ version 0.7.2.
