# Supplementary material for: A DNA barcode library for woody plants in tropical and subtropical China
Source: Sci Data. 2023 Nov 22;10:819. doi: 10.1038/s41597-023-02742-7 (PMC10665436; doi:10.1038/s41597-023-02742-7)
Supplement: Supplementary file 1 — Supplementary Table [file 41597_2023_2742_MOESM1_ESM.docx]

Supplementary Table 1. Details of collection locations for tropical and subtropical woody plants in China.

| Province | City | Standard barcode  samples | Standard barcode  species | Super barcode samples | Super barcode species | Latitude and longitude | Altitude (m) |
| --- | --- | --- | --- | --- | --- | --- | --- |
| Fujian | Fuzhou City | 40 | 40 | 1 | 1 | 26°03′N-26°9′N, 119°16′48″E-119°22′48″E | 42-237 |
|  | Nanping City | 189 | 182 | 10 | 10 | 26°34′48″N-26°43′12″N, 118°01′12″E-118°10′12″E | 115-879 |
| Guangdong | Lechang City | 8 | 8 | 0 | 0 | 25°10′12″N, 118°01′12″E-118°10′12″E | 299-624 |
|  | Shaoguan City | 42 | 42 | 42 | 42 | 24°42′36″N, 114°15′E | 793 |
|  | Zhaoqing City | 1144 | 534 | 193 | 188 | 23°10′12″N, 112°15′E | 350 |
| Guangxi | Chongzuo City | 318 | 215 | 0 | 0 | 21°36′N-23°22′N, 106°33′E-108°06′E | 200-220 |
|  | Guilin City | 353 | 330 | 6 | 6 | 25°42′N, 110°18′E | 150 |
|  | Hechi City | 106 | 106 | 106 | 106 | 25°07′48″N, 108°E | 547 |
| Guizhou | Anshun City | 3 | 3 | 0 | 0 | 25°21′N, 105°58′E | 800 |
|  | Bijie City | 8 | 8 | 0 | 0 | 27°10′48″N, 105°10′48″E | 1310 |
|  | Guiyang City | 10 | 10 | 0 | 0 | 26°21′30″N, 106°25′12″E | 1100 |
|  | Qiandongnan Miao and Dong Autonomous Prefecture | 1 | 1 | 0 | 0 | 26°34′48″N, 107°58′48″E | 600 |
|  | Qiannan Buyi and Miao Autonomous Prefecture | 152 | 148 | 3 | 3 | 25°24′36″N, 107°53′24″E | 426-812 |
|  | Qianxinan Buyi and Miao Autonomous Prefecture | 63 | 60 | 1 | 1 | 25°10′12″N, 106°19′48″E | 1186 |
|  | Tongren City | 33 | 32 | 3 | 3 | 27°43′12″N, 109°10′48″E | 459 |
|  | Zunyi City | 4 | 3 | 1 | 1 | 27°49′N, 106°55′48″E | 800-1300 |
| Hubei | Wuhan City | 297 | 262 | 7 | 7 | 30°31′48″N, 114°25′12″E | 427 |
| Hunan | Hengyang City | 90 | 85 | 2 | 2 | 27°04′N-27°20′N, 112°34′E-112°44′E | 96-1300 |
|  | Zhangjiajie City | 164 | 163 | 164 | 163 | 29°46′12″N, 110°05′24″E | 1401 |
| Jiangsu | Nanjing City | 237 | 219 | 8 | 8 | 32°03′N, 118°49′12″E | 48 |
| Jiangxi | Ganzhou City | 228 | 203 | 3 | 3 | 24°34′48″N-26°N, 114°03′E-118°28′12″E | 201-1304 |
| Shaanxi | Ankang City | 156 | 148 | 2 | 2 | 31°42′N-33°49′N, 108°01′E-110°01′E | 1508 |
|  | Baoji City | 13 | 13 | 0 | 0 | 34°22′12″N, 107°07′48″E | 618 |
| Yunnan | Chuxiong Yi Autonomous Prefecture | 60 | 60 | 60 | 60 | 24°32′24″N, 101°18′E | 2500 |
|  | Xishuangbanna Dai Autonomous Prefecture | 461 | 459 | 461 | 459 | 21°37′12″N, 101°33′36″E | 789 |
| Zhejiang | Hangzhou City | 401 | 383 | 13 | 13 | 30°15′N, 120°06″E | 18-52 |
|  | Lishui City | 28 | 28 | 28 | 28 | 27°45′36″N-27°52′48″N, 119°12′E | 1527 |
|  | Quzhou City | 125 | 125 | 125 | 125 | 29°15′N, 118°7′12″E | 581 |

Supplementary Table 2. The information of primer pairs used for PCR amplification

|  | Primers | Primer sequences (5'-3') | References |
| --- | --- | --- | --- |
| *rbcL* | rbcLa-F | ATGTCACCACAAACAGAGACTAAAGC | (Kress et al., 2009) |
|  | rbcLa-R | GTAAAATCAAGTCCACCRCG |  |
| *matK* | Kim_3F | CGTACAGTACTTTTGTGTTTACGAG | Kim, unpublished |
|  | Kim_1R | ACCCAGTCCATCTGGAAATCTTGGTTC |  |
|  | xF | TAATTTACGATCAATTCATTC | (Ford et al., 2009) |
|  | 5r | GTTCTAGCACAAGAAAGTCG |  |
|  | Gym_F1A | ATYGYRCTTTTATGTTTACARGC | (Li et al., 2011b) |
|  | Gym_R1A | TCAYCCGGA RATTTTGGTTCG |  |
| ITS | ITS-Leu | GTCCACTGAACCTTATCATTTAG | (Baum et al., 1998) |
|  | ITS4 | TCCTCCGCTTATTGATATGC | (White et al. 1990) |
|  | ITS5 | CCTTATCATTTAGAGGAAGGAG | (Stanford et al., 2000) |
| ITS2 | S2F | ATGCGATACTTGGTGTGAAT | (Chen et al., 2010) |
|  | S3R | GACGCTTCTCCAGACTACAAT |  |
